# Development of an implementation strategy for a workplace smoking cessation program with financial incentives using Implementation Mapping

**DOI:** 10.1371/journal.pone.0336553

**Published:** 2026-09-18

**Authors:** Floor A. van den Brand, Tessa Magnée, Onno C. P. van Schayck, Gera E. Nagelhout

**Affiliations:** 1 Department of Family Medicine (CAPHRI), Faculty of Health, Medicine & Life Sciences, Maastricht University, Maastricht, The Netherlands; 2 Netherlands Institute for Health Services Research, Utrecht, the Netherlands; 3 Department of Health Promotion (CAPHRI), Faculty of Health, Medicine & Life Sciences, Maastricht University, Maastricht, The Netherlands; 4 Centre of Expertise Perspective in Health, Avans University of Applied Sciences, Breda, The Netherlands; University of Nigeria - Enugu Campus, NIGERIA

## Abstract

**Introduction:**

Workplace group training programs for smoking cessation that incorporate financial incentives are cost-effective but face implementation challenges. This study aimed to systematically develop an evidence-informed implementation strategy for such programs in Dutch companies.

**Methods:**

From January 2019 to September 2021, we used Implementation Mapping, guided by the RE-AIM Framework, to design the strategy. A planning team comprising researchers, employers, employees, and smoking cessation counselors followed five structured steps: conducting a needs assessment; identifying adopters and implementers; setting implementation and maintenance outcomes; developing matrices of change objectives; producing protocols and materials; and creating an evaluation plan.

**Results:**

The needs assessment highlighted essential actions to enhance program implementation. Company directors and HR managers were identified as key adopters and implementers. Performance objectives included deciding to offer group training, applying financial incentives, proactively recruiting smoking employees, and organizing the intervention at least twice. Determinants influencing these objectives were attitude, outcome expectations, skills, self-efficacy, knowledge, normative beliefs, and perceived barriers. Evidence-based strategies were selected to address these determinants, and several supportive materials were developed: an implementation guide, informational website with downloadable resources, an animated promotional video, three testimonial videos, and a live webinar with recording to train employers in recruitment. The strategy was evaluated and refined based on stakeholder feedback.

**Conclusions:**

This study demonstrates how a systematic, theory-based approach can support the development of tailored implementation strategies for workplace smoking cessation programs with financial incentives. Engaging key stakeholders and addressing context-specific barriers and facilitators may strengthens the potential for real-world adoption and long-term sustainability. This approach can inform future implementation efforts for evidence-based tobacco control interventions in occupational settings.

## Introduction

Smoking remains a significant public health challenge, contributing to a wide range of chronic diseases and economic burdens [1,2]. Despite considerable efforts to reduce smoking rates, tobacco use persists and remains costly to both individuals and organizations, manifesting in health complications, absenteeism, and decreased productivity [3–6]. In the Netherlands 20.6% of the population smoked tobacco in 2021 [7], with smoking prevalence being higher among people with a low and moderate education than among highly educated individuals (23.9%, 24.2% and 15.3%, respectively). Although approximately one in three people who smoke make at least one serious quit attempt each year, the use of cessation support remains limited [7]. Smoking cessation medications combined with behavioral support are covered by Dutch health insurance; however, only 35% of individuals previously reported using any form of cessation assistance during their quit attempt [8].

These figures underline the need for accessible smoking cessation support, particularly of groups with a lower socioeconomic position. The workplace may be a promising setting for such support. In the Netherlands, employees have had the right to a smoke-free workplace since 1 January 2004, and smoke-free workplace legislation has since been strengthened, including the prohibition of indoor smoking rooms from 1 January 2021. The ban also applies to the hospitality sector and covers all tobacco products as well as e-cigarettes. In addition, the Dutch public campaign *Smoke-Free Generation* (*Rookvrije Generatie*) encourages smoke-free workplaces and grounds and supports companies in helping employees quit smoking, for example through cessation support or referral to professional services.

Employers can hold a critical role in promoting smoking cessation among their employees [9]. Workplace-based interventions, including group training programs, have demonstrated their effectiveness in encouraging employees to quit smoking [9–12]. Research further shows that incorporating financial incentives can amplify the success of smoking cessation programs and boost employee participation [13–17]. This is especially relevant for employees with a lower socioeconomic position (SEP), a group with higher smoking prevalence, who may benefit from greater access to evidence-based professional smoking cessation support through workplace interventions [15,18,19]. However, in many countries, workplace smoking cessation support is not yet embedded in routine company policy, and financial incentives are rarely used.

This evidence-to-practice gap reflects a broader implementation challenge: effective health interventions are often difficult to translate into routine workplace practice [20,21]. Successful implementation depends on selecting strategies that fit the specific context and address relevant barriers [22–24]. Previous research has revealed numerous barriers to the successful implementation of workplace health programs [15,22,25]. A review identified over 50 different implementation barriers and facilitators for workplace health promotion programs [26]. The most commonly cited barriers and facilitators were found at the organizational level. Among these, strong (upper) management support for the intervention was a significant facilitator of successful health program implementation. Similarly, compatibility between the health intervention and the organization’s structure and culture was also a frequently cited enabler. In addition, the most common barriers included a lack of financial, staffing, or material resources and a lack of perceived management support by implementers on site.

High participation rates are essential for the effectiveness of workplace health promotion programs, yet reaching and engaging employees, particularly those with a lower SEP, remains a challenge [27]. Communication about these programs may not always reach these employees effectively, and even when it does, these employees may still lack the motivation to participate [26]. A review found that programs that appealed to blue-collar workers tended to feature group activities, low effort requirements, affordability, and accessibility in terms of time and location, while shift work, limited job flexibility, and the belief that employers should not have a role in their health and lifestyle were identified as barriers to participation [15].

Financial incentives may help address participation challenges [12,28,29]. In a large study, incentives increased employee enrollment in a smoking cessation program from 5.4% to 15.4% and program completion from 2.5% to 10.8% [30]. Similar positive effects on smoking treatment engagement have been reported in lower-SEP populations [14,31–33]. However, implementation of financial incentives may be complicated by concerns about costs and public acceptability [17,34–36].

A structured approach is therefore needed to develop implementation strategies that are responsive to these contextual barriers. Implementation Mapping (IM) is one such approach, providing a systematic process for designing theory- and evidence-informed strategies to support the adoption, implementation, and maintenance of interventions [37]. It encompasses five tasks: (1) conducting a needs assessment to identify program adopters and implementers; (2) specifying adoption and implementation outcomes and performance objectives, identifying determinants, and creating matrices of change objectives; (3) selecting theoretical change methods and designing or choosing implementation strategies; (4) developing implementation protocols and materials; and (5) evaluating implementation outcomes [37]. This study describes how we used Implementation Mapping to design implementation strategies and practical applications based on theory and evidence to facilitate the implementation of a workplace group smoking cessation program with financial incentives in workplaces in the Netherlands.

## Methods

### Setting and target group

In this study, we followed the Implementation Mapping process from January 2019 to September 2021 to develop an implementation strategy for a smoking cessation group training program with financial incentives that could be used by companies throughout the Netherlands. The strategy was specifically intended for implementation in companies with a high proportion of lower-SEP employees. We invited employers and employees from our professional networks to participate in the implementation planning group, including large organizations in the sheltered employment, healthcare, and public safety sectors, located across the Netherlands. This study adhered to ethical principles and Dutch National guidelines for conducting research and complied with the General Data Protection Regulation (GDPR). Participants provided their written informed consent to participate in the study.

### Implementation planning group

The implementation planning was led by a research team consisting of health scientists with expertise in tobacco control and smoking cessation, socioeconomic health disparities and health promotion. FvdB and TM were trained in Intervention Mapping. Prior to the project’s launch, an implementation planning group was formed, including five employers, two employees from different companies who smoke(d) (one moderate and one lower-SEP), three health consultancy organizations, an occupational health service, an expertise center for socioeconomic differences in health, a communication expert, and five health behavior scientists. The implementation planning group also included a major provider of in-company smoking cessation training and one of their smoking cessation counselors. This provider would apply the implementation strategy once it was developed, enabling the research team to test and evaluate it. Between September 2019 and January 2021, the research team facilitated six group meetings to collaboratively design (“co-create”), the implementation strategy. The first meeting was held in person; due to the COVID-19 pandemic, all subsequent meetings took place online via videoconference. After the first version of the website and materials were launched, we conducted in-person online or telephone interviews with our stakeholders and with employers and employees who smoke to evaluate the materials. For the website, we interviewed 10 employers and 7 stakeholders, for the Implementation planning guide we interviewed an additional 8 employers and 2 stakeholders, and for the posters, promotional animated video and testimonial video we interviewed 10 employers, 11 employees and 9 stakeholders (a total of 18 different employers, 11 employees and 9 stakeholders were interviewed). Based on their comments and suggestions, the materials were adapted, two new posters were designed, and additional materials were developed, including two testimonial videos, the webinar and related materials (webinar video recording, role-play video, factsheets, leaflet with communication tips).

### Theoretical framework

We employed the RE-AIM Framework [38] as our theoretical foundation. This framework helps translate research into practice and improves the likelihood of successful real-world interventions. RE-AIM stands for reach, effectiveness, adoption, implementation, and maintenance. Reach is defined as the absolute number, proportion, and representativeness of individuals willing to participate in the intervention. Effectiveness refers to the impact the intervention has on significant outcomes. As effectiveness of the current intervention has been established previously [13,39], this was not part of the current study. Adoption refers to the decision to use the intervention within the organization. Implementation means trying out the intervention to a point where it can be experienced and evaluated by the organization. Finally, maintenance refers to the extent to which the intervention is continued and integrated into the practices and policy of the organization [38].

### Implementation Mapping

Implementation Mapping provides a systematic process for developing strategies to improve the adoption, implementation, and maintenance of evidence-based interventions in real-world settings [23,37,40]. The Implementation Mapping process consisted of five tasks.

First, adopters and implementers of the intervention were identified and a needs assessment was conducted to identify barriers and facilitators for the implementation of a smoking cessation training with financial incentives in workplaces. As adopters and implementers, we identified company directors and managers involved in human resources and/or occupational health (such as human resources managers, health and safety advisors, occupational physicians and organizational vitality coaches); from here on referred to as ‘employers’. For the needs assessment, we conducted individual qualitative interviews with employers and employees.

We interviewed 18 employers of organizations in the Netherlands that employ relatively many employees with a lower socioeconomic position. In addition, we interviewed 19 employees who currently or formerly smoked, of whom some had participated in the workplace smoking cessation training with or without financial incentives and of whom some had not participated in a workplace smoking cessation training. The results and details of this study can be found in previous publications [41,42]. Additionally, we searched PubMed and the Cochrane Database of Systematic reviews for systematic reviews on the implementation of health promotion and smoking cessation programs with or without incentives in the workplace or other settings. We used (combinations of) several search terms, including “implementation”, “health promotion”, “smoking”, “tobacco”, and “incentives”. We did not restrict our search strategy to the workplace setting, because we expected the evidence in this setting to be scarce, and findings from other settings to be potentially relevant as well. We wrote a narrative summary of the studies that we identified.

For the second task of Implementation Mapping, based on the needs assessment, scientific literature and discussions with the stakeholders, we determined outcomes and performance objectives for program use. This means that we identified who needed to do what to achieve the intended goals for the reach, adoption, implementation and maintenance of the intervention. Then, a matrix was created in which for all performance objectives, determinants of change were identified that were likely to influence them, and change objectives were formulated.

For the third task, we selected theory-based methods to influence the determinants that were previously identified, and we designed implementation strategies. In the fourth task, in co-creation with the stakeholder group, we produced implementation protocols and materials, including a website, several video’s, an online communication training, a planning and communication guide for employers, and posters. Between April and June 2020, the initial versions of the materials were further improved based on feedback that was collected through individual interviews with planning group members (n = 12) and employers (n = 20). The posters were evaluated and adapted based on interviews with employees who were (former) smokers (n = 11). For the fifth and last task, we established a plan for the evaluation of the implementation outcomes.

## Results

The outcomes of the five tasks of the Implementation Mapping process [37] that were conducted as part of the implementation planning in the current study are described below.

### Task 1: Conduct a needs assessment

We conducted interviews with employers to identify perceived barriers and facilitators for the reach, adoption, implementation, and maintenance of a workplace smoking cessation training with financial incentives (published elsewhere [42]). In interviews with employees, we explored how employees who smoke can best be reached and stimulated to participate, which practical barriers and facilitators affect participation, and how the training can be maintained within the organization (published elsewhere [41]). Table 1 presents the key actions identified from these interviews. Overall, the findings showed that successful implementation requires proactive communication and manager training to improve reach, attention to fairness and clear communication about benefits to support adoption, accessible delivery of the training to facilitate implementation, and integration of smoking cessation support into organizational vitality policy to support maintenance.

**Table 1:** Key actions required to improve the implementation of workplace smoking cessation group trainings with financial incentives, as identified from qualitative interviews with employers and employees conducted as part of the needs assessment.

	Employer interviews	Employee interviews
**Reach**	Train managers in how to effectively engage their employees and how to encourage them to enroll in the smoking cessation training.	Use pro-active communication strategies such as word-of-mouth to inform about and promote the smoking cessation training.
		Train managers to discuss with and stimulate employees who smoke to participate.
**Adoption**	Assure employers that non-smoking employees will not perceive the incentives as unjust, or alternatively, provide options for different incentives that are viewed as more equitable.	Explain to all employees why the smoking cessation training is being offered and the benefits it can have for all staff.
**Implementation**	Explain the cost-effectiveness of smoking cessation group trainings combined with financial incentives.	Make the training as accessible as possible by reimbursing time spent and offering the program at the workplace or nearby.
**Maintenance**	Integrate the smoking cessation training into organizational vitality policy.	Integrate the smoking cessation training into organizational vitality policy.

### Task 2: Identify adoption and implementation outcomes, performance objectives, determinants and change objectives

**Fig 1:**
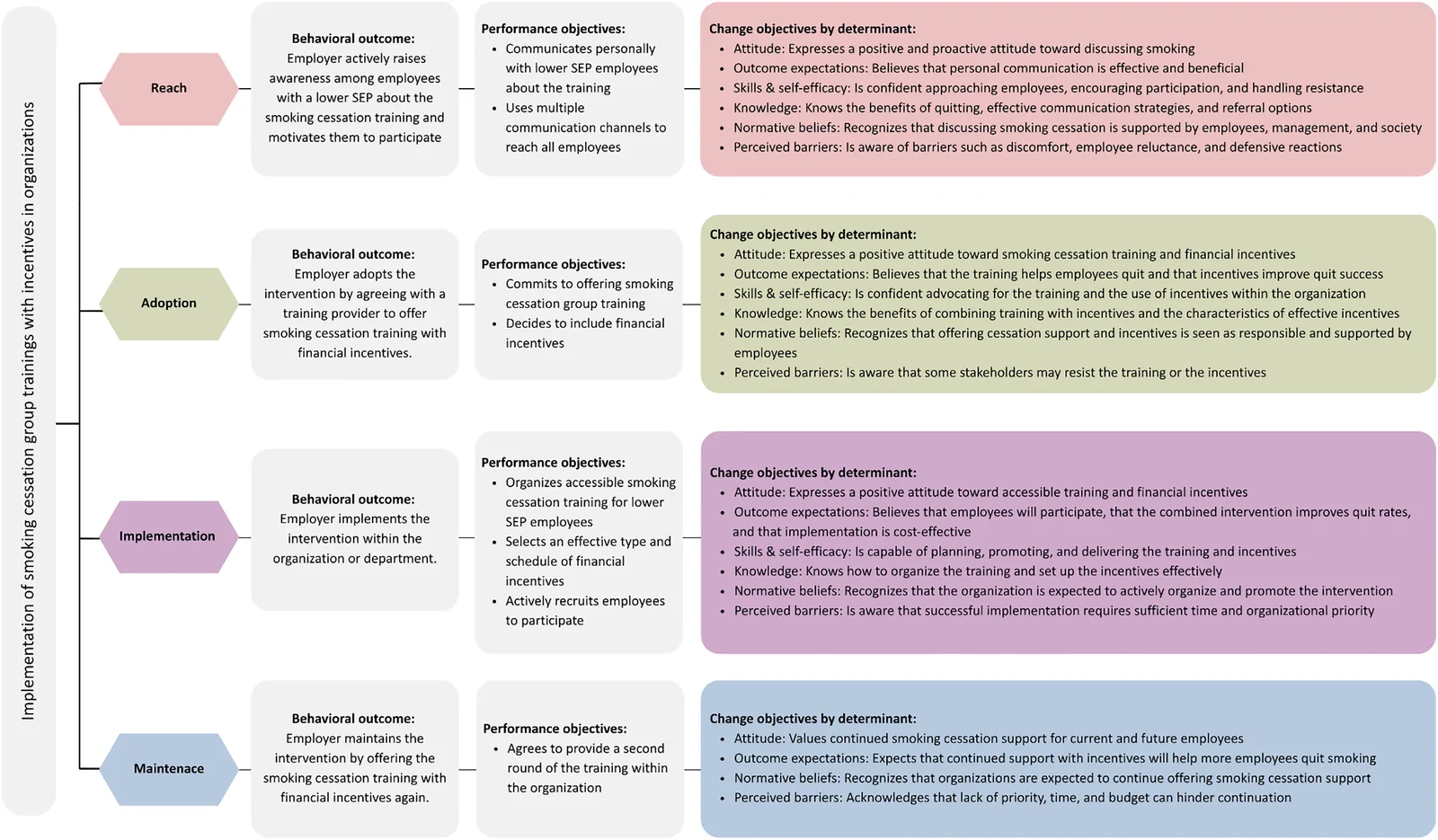
Summarized overview of behavioral outcomes, performance objectives and change objectives to promote the implementation of smoking cessation group trainings with financial incentives in organizations.

We identified employers as the main actors in implementation. We defined relevant behavioral outcomes, performance objectives and change objectives for the RE-AIM components, creating a matrix of change, which is fully presented in S1 File and summarized in Fig 1. For reach, the main objectives were to encourage employers to actively promote the training, particularly through proactive in-person communication and the use of multiple communication channels. For adoption, the focus was on employers’ commitment to offering the training and deciding to include financial incentives. For implementation, employers were expected to organize the training in an accessible way, select an appropriate incentive structure, and actively recruit employees. For maintenance, employers should continue to offer the training in the future. We selected the following determinants: attitude, skills & self-efficacy and normative beliefs (Theory of Planned Behavior [43]); Knowledge and outcome expectations (Social Cognitive Theory [44]) and perceived barriers (Health Belief Model [45]).

### Task 3: Select theoretical change methods and design implementation strategies

Based on the literature [23,46], we selected theoretical methods for behavioral change suitable for our implementation strategy and translated these into practical applications and materials. Tables 2–4 summarize the methods selected for each determinant and RE-AIM stage. For reach, change methods addressed employers’ attitudes, outcome expectations, skills and self-efficacy, knowledge, normative beliefs, and perceived barriers. Practical applications focused on promoting proactive personal communication, strengthening confidence and communication skills, providing information, addressing resistance, and reframing the employer’s role as offering support. For adoption, change methods targeted employers’ attitudes, outcome expectations, skills and self-efficacy, knowledge, normative beliefs, and perceived barriers regarding smoking cessation training with financial incentives. Practical applications focused on communicating effectiveness, fairness, health and cost-benefit arguments, providing examples from other organizations, and reframing incentives as rewarding healthy behavior. For implementation and maintenance, change methods mainly targeted attitudes, outcome expectations, normative beliefs, and perceived barriers. Practical applications included sharing feedback on participant experiences and quit outcomes, showing approval from other employers and employees, and providing practical support for planning, organization, and continuation of the intervention over time.

**Table 2:** Implementation strategies developed to enhance the reach of smoking cessation group trainings with financial incentives in organizations.

Reach				
Determinants	Theoretical Change Methods	Definition*	Practical Applications	Materials
Attitude	Belief selection	Using messages designed to strengthen positive beliefs, weaken negative beliefs, and introduce new beliefs.	Strengthening the beliefs that approaching employees is positive, necessary and easy to perform. Weakening the beliefs that approaching employees is negative, ‘nagging’, intrusive, difficult	Webinar part 1, 2, 3
	Shifting perspective	Encouraging taking perspective of the other.	Employees show their experience with and viewpoint on talking about smoking with their manager.	Webinar part 2, Testimonial video line manager
Outcome expectations	Persuasive communication	Guiding individuals toward the adoption of an idea, attitude or action by using arguments or other means.	Show that a personal communication approach is necessary to convince employees to participate in a smoking cessation training.	Webinar part 1, 3, Implementation planning guide, Website
	Scenario-based risk information	Providing information that may aid the construction of an image of the ways in which a future loss or accident might occur	A scenario is presented of a peer organization that failed to personally recruit employees and this led to very low enrolment numbers.	Webinar part 1
Skills & self-efficacy	Modeling	Providing an appropriate model; being reinforced for the desired action.	Show an employer who has successfully promoted smoking cessation to his/her employees.	Webinar part 1, Testimonial video line manager
	Active learning, Guided practice	Encouraging learning from goal-driven and activity-based experience, Prompting individuals to rehearse and repeat the behavior various times, discuss the experience and provide feedback.	Interactive role-play. The trainer models the way to approach and speak with employees about smoking and then asks the employers to practice together.	Webinar part 2, Role-play video with actors
Knowledge	Conveying information	Providing information about a topic.	Providing facts, data and recommendations regarding smoking cessation group trainings, financial incentives, and effective communication.	Website, Implementation planning guide, Communication factsheet
Normative beliefs	Provide opportunities for social comparison	Facilitating observation of non-expert others in order to evaluate one’s own opinions and performance abilities.	Show that other (‘role-model’) organizations actively promote smoking cessation among their employees.	Webinar part 1
	Planned coping Responses	Prompting participants to list potential barriers and ways to overcome these.	Let employers think about potential barriers, on how to react if employees are not interested in smoking cessation, on how to interpret the fact that employees will relapse.	Webinar part 1,2,3, Role-play video with actors
Perceived Barriers	Shifting focus	Prompting hiding of the unpopular behavior or shifting attention away from the behavior (toward a new reason for performing the behavior).	The employer is not ‘telling employees to quit smoking’ but instead ‘offers the opportunity to get support if they want it’. The employer is not ‘interfering in the employee’s private life’ but instead ‘shows interest in the needs and wellbeing of the employee’.	Webinar part 2,3

* Definitions as described by: Eldredge LKB, Markham CM, Ruiter RA, Fernández ME, Kok G, Parcel GS. *Planning health promotion programs: an intervention mapping approach*. John Wiley & Sons; 2016.

**Table 3:** Implementation strategies developed to enhance the adoption of smoking cessation group trainings with financial incentives in organizations.

Adoption				
Determinants	Theoretical Change Methods	Definition	Practical Applications	Materials
Attitude	Arguments	Using a set of one or more meaningful premises and a conclusion.	Provide arguments about the benefits of providing smoking cessation trainings and combine these with financial incentives for quit success (employer responsibility, employee health outcomes, cost-benefit, and effectiveness).	Website, Implementation planning guide, Promotional animated video, Testimonial video’s
	Belief selection	Using messages designed to strengthen positive beliefs, weaken negative beliefs, and introduce new beliefs.	Conveying the message that financial incentives are not unfair, that employers are not rewarding smokers but that they are rewarding employees who have quit smoking. In other words, they are rewarding healthy behavior.	Website, Implementation planning guide, Promotional animated video
Outcome expectations	Persuasive communication	Guiding individuals toward the adoption of an idea, attitude or action by using arguments or other means.	Provide research data to show that adding financial incentives to a smoking cessation training significantly increases quit success.	Website, Promotional animated video
Skills & self-efficacy	Verbal persuasion	Using messages that suggest that the participant possesses certain capabilities.	Show examples of other (comparable) employers/organizations that were able to successfully implement the intervention.	Website, Testimonial video’s
Knowledge	Conveying information	Providing information about a topic.	Provide statistics about the positive outcomes of adopting a smoking cessation training; Provide information about the characteristics of effective financial incentives.	Website, Implementation planning guide
Normative beliefs	Information about others’ approval	Providing information about what others think about the person’s behavior and whether others will approve of any proposed behavior change.	Show that employees are positive about being offered a smoking cessation training with financial incentives. Show that other employers also provide smoking cessation trainings with financial incentives.	Webinar part 2, Testimonial video’s
Perceived Barriers	Shifting focus	Prompting hiding of the unpopular behavior or shifting attention away from the behavior (toward a new reason for performing the behavior).	Show that employees approve of providing smoking cessation trainings with financial incentives; Shift the narrative from ‘rewarding smokers’ (unfair) to ‘rewarding people for not smoking’ (fair) and to ‘being a good employer who promotes a healthy organization’.	Website, Promotional animated video, Testimonial video’s

**Table 4:** Implementation strategies developed to enhance the implementation and maintenance of smoking cessation group trainings with financial incentives in organizations.

Implementation and Maintenance				
Determinants	Theoretical Change Methods	Definition	Practical Applications	Materials
Attitude	Direct experience	Encouraging a process whereby knowledge is created through the interpretation of experience.	Providing the employer with feedback from employees who participated in the initial group training that was conducted within the organization.	Standard report from smoking cessation provider
Outcome expectations	Feedback	Giving information to individuals regarding the extent to which performance is having an impact	Providing the employer feedback on smoking cessation rates from the initial group training that was conducted within the organization.	Standard report from smoking cessation provider
Normative beliefs	Information about other’s approval	Providing information about what others think about the behavior and whether others will approve or disapprove of the proposed behavior	Providing testimonials from other employers and employees about their approval of organizations facilitating smoking cessation trainings with financial incentives.	Testimonial video’s
Perceived Barriers	Verbal persuasion	Using messages that suggest that the participants possess certain capabilities.	Providing an easy-to-use schedule for the planning and organization of smoking cessation trainings. Providing information that the costs of smoking cessation counseling are reimbursed by health insurance providers for each individual once every year.	Implementation planning guide, Website

### Task 4: Produce implementation protocols and materials

In close collaboration with content developers and communication experts, we developed a corporate design, including a logo, color scheme, and fonts. We named the implementation strategy *Quit Stronger Together*. We developed the following practical applications: a website, an animated video on effectiveness of the intervention, two testimonial videos with success stories of employers and employees, a video with two actors demonstrating how to talk about quitting smoking, an implementation protocol for employers, an online training for employers, and recruitment posters aimed at employees (Table 5).

**Table 5:** Overview of developed materials.

Developed material	Description
Logo design	Logo with slogan ‘Quit Stronger Together’/ ‘Samen Sterker Stoppen’.
Website	Employer website with information, tools, videos, posters, FAQs, and links.
Implementation planning guide	Employer guide with planning, recruitment, communication, incentive, and template materials.
Promotional animated video	Short animated video on workplace cessation trainings with financial incentives.
Testimonial video 1 (waste management)	Video with blue-collar workers’ experiences and researcher input on incentives.
Testimonial video 2 (supermarket chain)	Video with a branch manager and employee sharing experiences with training and incentives.
Testimonial video 3 (line manager)	Video on using personal contact and follow-up to encourage participation.
Recruitment posters	Poster templates for recruiting employees, with or without mentioning incentives.
Webinar	Webinar on motivating employees through examples, role-play, and communication guidance.
Role play video: ‘talking about quitting smoking’	Video demonstrating effective and ineffective conversations about quitting smoking.
Communication factsheet	Factsheet with tips and example sentences for discussing smoking cessation.
Factsheet on tax regulations	Factsheet on tax rules and financing cessation training and incentives.
Telephone script for smoking cessation provider	Provider script for explaining financial incentives to employers.
Leaflet with communication tips for smoking cessation provider	Provider leaflet with incentive information, FAQs, and persuasive wording.

### Task 5: Evaluate implementation outcomes

For Task 5, we have planned a comprehensive evaluation of the implementation outcomes, utilizing the RE-AIM framework [47] and key process evaluation components identified by Linnan & Steckler (2004) [48]. To test the implementation strategy, the provider of in-company smoking cessation training programs from our implementation group integrates the strategy into their communications with companies considering a workplace smoking cessation group training. The goal is to encourage these companies to implement the training in combination with financial incentives and employ active, in-person communication strategies to recruit employees from lower SEP. To evaluate the strategy, we identify facilitators and barriers across the RE-AIM dimensions, with the exception of effectiveness, which has been previously established. Using the matrices of change objectives, we assess whether the implementation strategies effectively influenced the targeted determinants of behavior change.

From the employers’ perspective, we measure reach by assessing the number and proportion of employees participating in the smoking cessation trainings and determining whether this aligns with employers’ expectations. We also examine what factors helped or hindered employers in reaching employees with a lower SEP. For adoption, we measure the proportion of employers who opted to implement financial incentives for smoking cessation success when ordering training from the provider, and explore the factors that influenced these decisions. Additionally, we assess the level of organizational support for adopting the smoking cessation training with financial incentives. Regarding implementation, we evaluate the extent to which employers utilized the provided implementation materials and engaged in active, in-person communication strategies. For maintenance, we assess whether organizations that conducted an initial smoking cessation training commit to organizing a second training within six months.

To collect these data, we use monitoring data from the smoking cessation provider and conduct shorter telephone surveys as well as longer qualitative interviews with employers who have organized a smoking cessation training. Additionally, we follow up with these companies six months post-training to determine if a subsequent training has been scheduled.

We also evaluate the role of the smoking cessation provider in the implementation process. This includes assessing the communication channels they used to recruit companies and evaluating the fidelity, dose delivered [48], and barriers and facilitators in utilizing the implementation strategy. Moreover, we investigate contextual influences and determine the provider’s willingness to continue using the communication strategy after the study period concludes. These outcomes are assessed through qualitative interviews with the provider’s account managers. The results of this evaluation will be published elsewhere.

## Discussion

This study described the systematic development of an implementation plan to promote the widespread adoption of the evidence-based intervention *Quit Stronger Together*, a workplace smoking cessation group training combined with financial incentives. A major focus of our implementation strategy was to increase reach. We designed approaches that help employers engage employees with a lower SEP and motivate them to participate in the training. Our needs assessment revealed that both employers and employees who smoke preferred a proactive, in-person approach to encourage enrollment. This is in line with earlier findings showing that passive promotion is often insufficient in workplace health promotion and that participation among lower-SEP employees is shaped by accessibility, communication style, and workplace culture [15,26,27]. However, employers often lacked the confidence and skills to effectively communicate with employees about smoking. We therefore developed a live webinar training for managers, equipping them with the communication skills necessary to engage employees. The implication is that employers should not simply be expected to promote smoking cessation support on their own, but may need specific training and tools to do so effectively and without creating resistance or stigma [49,50].

A second important finding concerns the adoption of financial incentives. Although financial incentives can improve treatment engagement and quit outcomes, employers may hesitate because of concerns about fairness, feasibility, and costs [42,51,52]. Our implementation strategy therefore included messages and materials aimed at strengthening positive attitudes toward incentives and reframing them as support for healthy behavior rather than as unfair rewards for smokers [34]. We crafted messaging that highlighted the effectiveness of financial incentives in promoting quit success, outlined the associated costs and benefits, featured role models, emphasized the employer’s responsibility for employee health, and addressed common concerns regarding the perceived unfairness of financial incentives for non-smoking employees, offering clear explanations and responses to these remarks. At the same time, we encountered a broader implementation challenge: how to balance fidelity to an evidence-based intervention with adaptation to local organizational preferences. Employers expressed a preference for flexibility in the type, amount, or schedule of financial incentives [42]. Since the existing evidence does not define the optimal parameters for incentives [17], we opted to retain the original form as much as possible while providing guidance on key characteristics that may contribute to incentive effectiveness such as ensuring the incentives are substantial, attractive and directly tied to successful quitting outcomes [17,53,54].

These findings have implications for both implementation practice and health equity. Scaling up workplace smoking cessation support requires more than evidence of effectiveness alone. Employers also need practical tools, communication guidance, and organizational arguments that address ethical as well as operational concerns [50,52]. This may be particularly important to reach employees with a lower SEP, among whom smoking prevalence is often higher and participation barriers may be greater [15]. Our findings suggest that participation is influenced not only by whether support is offered, but also by how, by whom, and under what practical conditions it is introduced. This underlines the importance of implementation strategies that are sensitive to literacy, workplace culture, shift work, and other structural constraints that may disproportionately affect lower-SEP employees [15,55].

Although this study was conducted in the Netherlands and the developed materials are therefore context-specific, the broader implementation logic is likely to be transferable. The feasibility of adoption is partly shaped by national tax regulations, financing arrangements, occupational health structures, and employer responsibilities, which will differ across countries [24]. However, the broader challenges identified in this study; limited reach, the importance of proactive and personal communication, employer hesitation about financial incentives, and the need to tailor strategies to organizational routines, are not unique to the Dutch context. The main recommendation for other settings is therefore not that the final implementation package should be copied unchanged, but that the structured process of Implementation Mapping can be used to develop context-sensitive implementation strategies that fit local workplace, policy, and financing conditions.

### Strengths and limitations

One of the strengths of this research was the involvement of a large planning group that included key stakeholders such as employers and employees who smoke, communication professionals, and an interdisciplinary group of researchers. Their contributions offered critical insights into the needs of organizations, leading to creative and tailored implementation strategies. Furthermore, collaboration with the smoking cessation provider ensured their enthusiasm for the plan and commitment to testing it in practice. Contributions from experts in communication, health promotion, addiction, and smoking cessation counseling further enhanced the identification of effective strategies and the design of appropriate materials. Another strength of our study was that we employed an iterative co-creation process, in which initial strategies and materials were developed and refined based on ongoing feedback from our stakeholder group, employers and employees. For example, we made substantial changes in the recruitment posters for the smoking cessation training after evaluating them with employees. In addition, because of feedback from employers we added a factsheet with information on Dutch tax regulations and guidance on financing smoking cessation trainings and financial incentives. Despite its strengths, the study also faced challenges, primarily related to limited budget and time. These constraints affected the development of additional implementation materials that could have enhanced the intervention, particularly in the maintenance stage. For instance, while promoting smoke-free workplaces may encourage participation and foster a non-smoking culture, such broader environmental changes were not targeted within this study. Similarly, we did not focus on selecting strategies to facilitate integration of the cessation program into corporate health plans or to shift organizational values. Efforts centered on training managers to encourage employee participation but did not extend to guiding them on allocating time and resources or involving colleagues in recruitment. In addition, our implementation strategy aimed to persuade employers to adopt financial incentives for smoking cessation, but a complementary approach could involve securing coverage for these incentives through health insurers. Despite discussions we had with health insurers, gaining their support proved challenging. In the future, stronger advocacy efforts may be needed to encourage insurers to cover these incentives, making them more accessible and sustainable. The study also encountered challenges related to the COVID-19 pandemic. Social distancing measures and remote work necessitated a shift from in-person communication training for employers to a webinar format. While this adaptation allowed us to reach a larger number of employers, it may have also reduced the training’s effectiveness, as the online format provided less opportunity for employers to practice their communication skills. Another consequence of the pandemic was that in-person group training sessions became unfeasible during the testing period, leading to a significant decline in companies requesting smoking cessation training from the provider. As a result, we had to adjust our approach to inform the refinement of the implementation strategy. Since comparing the number of companies implementing financial incentives and employee participation rates before and after the communication strategy’s implementation was no longer scientifically viable, we instead relied solely on qualitative data to improve the implementation strategy. This involved conducting online interviews with employers, employees, and stakeholders. This change in approach may have influenced the final implementation strategy. The final evaluation of the effectiveness of the implementation strategy is currently underway and will be published in another paper.

## Conclusion

Using the Implementation Mapping approach, we developed a theory- and evidence-informed implementation plan to support the wider uptake of workplace smoking cessation group training with financial incentives. Through a co-creation process involving employers, employees who smoke, communication professionals, researchers, and a smoking cessation provider, we identified key implementation outcomes and determinants, selected implementation strategies, and developed practical materials to support delivery. Our findings show that successful implementation depends not only on intervention effectiveness, but also on proactive and tailored recruitment, managerial communication skills, and organizational readiness to adopt financial incentives. Although some components of the plan require adaptation to local workplace structures, financing arrangements, and policy environments, the systematic approach used in this study may provide a useful template for implementing similar workplace smoking cessation interventions in other contexts and countries.

## Supporting information

S1 File Matrices of change objectives for the implementation of smoking cessation group trainings with financial incentives in organizations. (DOCX)

## References

[1] USDHHS. The health consequences of smoking-50 years of progress. a report of the Surgeon General. Atlanta (GA): US Department of Health and Human Services, Centers for Disease Control and Prevention, National Center for Chronic Disease Prevention and Health Promotion, Office on Smoking and Health; 2014.

[2] Goodchild M, Nargis N, Tursan d’Espaignet E. Global economic cost of smoking-attributable diseases. Tob Control. 2018;27(1):58–64. doi: 10.1136/tobaccocontrol-2016-053305 28138063 PMC5801657

[3] Max W. The financial impact of smoking on health-related costs: a review of the literature. Am J Health Promot. 2001;15(5):321–31. doi: 10.4278/0890-1171-15.5.321 11502013

[4] Weng SF, Ali S, Leonardi-Bee J. Smoking and absence from work: systematic review and meta-analysis of occupational studies. Addiction. 2013;108(2):307–19. doi: 10.1111/add.12015 23078132

[5] Halpern MT, Shikiar R, Rentz AM, Khan ZM. Impact of smoking status on workplace absenteeism and productivity. Tob Control. 2001;10(3):233–8. doi: 10.1136/tc.10.3.233 11544387 PMC1747570

[6] Troelstra SA, Coenen P, Boot CR, Harting J, Kunst AE, van der Beek AJ. Smoking and sickness absence: a systematic review and meta-analysis. Scand J Work Environ Health. 2020;46(1):5–18. doi: 10.5271/sjweh.3848 31478055

[7] Van Aerde MB, Willemsen J. Kerncijfers roken 2023-2024; 2023.

[8] van Westen-Lagerweij NA, Bommelé J, Willemsen MC, Croes EA. Mentioning smoking cessation assistance during healthcare consultations matters: findings from Dutch survey research. Eur J Public Health. 2022;32(5):747–52. doi: 10.1093/eurpub/ckac106 36001051 PMC9527971

[9] Cahill K, Lancaster T. Workplace interventions for smoking cessation. Cochrane Database Syst Rev. 2014;2014(2):CD003440. doi: 10.1002/14651858.CD003440.pub4 24570145 PMC11285308

[10] Stead LF, Carroll AJ, Lancaster T. Group behaviour therapy programmes for smoking cessation. Cochrane Database Syst Rev. 2017;3(3):CD001007. doi: 10.1002/14651858.CD001007.pub3 28361497 PMC6464070

[11] Ayaz D, Asi E, Meydanlioglu A, Oncel S. Effectiveness of smoking cessation interventions in the workplace: a systematic review and meta-analysis. Am J Ind Med. 2024;67(8):712–22. doi: 10.1002/ajim.23627 38884628

[12] Cahill K, Lancaster T. Workplace interventions for smoking cessation. Cochrane Database Syst Rev. 2014;2014(2):CD003440. doi: 10.1002/14651858.CD003440.pub4 24570145 PMC11285308

[13] van den Brand FA, Nagelhout GE, Winkens B, Chavannes NH, van Schayck OCP. Effect of a workplace-based group training programme combined with financial incentives on smoking cessation: a cluster-randomised controlled trial. Lancet Public Health. 2018;3(11):e536–44. doi: 10.1016/S2468-2667(18)30185-3 30342875

[14] Kendzor DE, Businelle MS, Frank-Pearce SG, Waring JJC, Chen S, Hébert ET, et al. Financial incentives for smoking cessation among socioeconomically disadvantaged adults: a randomized clinical trial. JAMA Netw Open. 2024;7(7):e2418821. doi: 10.1001/jamanetworkopen.2024.18821 38954415 PMC11220567

[15] Damen MAW, Detaille SI, Robroek SJW, Engels JA, de Lange AH. Factors associated with blue-collar workers’ participation in Worksite Health Promotion Programs: a scoping literature review. Health Promot Int. 2023;38(3):daad052. doi: 10.1093/heapro/daad052 37379570 PMC10306361

[16] Tappin D, Lee J, McConnachie A, Kock L, Higgins ST, Heil SH, et al. Financial rewards for smoking cessation during pregnancy and birth weight: a meta-analysis. JAMA Netw Open. 2025;8(3):e250214. doi: 10.1001/jamanetworkopen.2025.0214 40048164 PMC11886724

[17] Notley C, Gentry S, Livingstone-Banks J, Bauld L, Perera R, Conde M, et al. Incentives for smoking cessation. Cochrane Database Syst Rev. 2025;1(1):CD004307. doi: 10.1002/14651858.CD004307.pub7 39799985 PMC11725379

[18] Van den Brand FA, Dohmen LME, Van Schayck OCP, Nagelhout GE. “Secretly, it’s a competition”: a qualitative study investigating what helped employees quit smoking during a workplace smoking cessation group training programme with incentives. BMJ Open. 2018;8(11):e023917. doi: 10.1136/bmjopen-2018-023917 30478122 PMC6254401

[19] Theodoulou A, Fanshawe TR, Leavens E, Theodoulou E, Wu AD, Heath L, et al. Differences in the effectiveness of individual-level smoking cessation interventions by socioeconomic status. Cochrane Database Syst Rev. 2025;1(1):CD015120. doi: 10.1002/14651858.CD015120.pub2 39868569 PMC11770844

[20] Glasgow RE, Vinson C, Chambers D, Khoury MJ, Kaplan RM, Hunter C. National Institutes of Health approaches to dissemination and implementation science: current and future directions. Am J Public Health. 2012;102(7):1274–81. doi: 10.2105/AJPH.2012.300755 22594758 PMC3478005

[21] Morris ZS, Wooding S, Grant J. The answer is 17 years, what is the question: understanding time lags in translational research. J R Soc Med. 2011;104(12):510–20. doi: 10.1258/jrsm.2011.110180 22179294 PMC3241518

[22] Wolfenden L, Goldman S, Stacey FG, Grady A, Kingsland M, Williams CM, et al. Strategies to improve the implementation of workplace-based policies or practices targeting tobacco, alcohol, diet, physical activity and obesity. Cochrane Database Syst Rev. 2018;11(11):CD012439. doi: 10.1002/14651858.CD012439.pub2 30480770 PMC6362433

[23] Eldredge LKB, Markham CM, Ruiter RA, Fernández ME, Kok G, Parcel GS. Planning health promotion programs: an intervention mapping approach. John Wiley & Sons; 2016.

[24] Lipman SA, de Buisonjé DR, van der Swaluw K. Promoting healthy behaviour with financial incentives: three challenges and solutions for large scale implementation. Eur J Health Econ. 2026. doi: 10.1007/s10198-025-01893-1 41632413 PMC13619794

[25] Rojatz D, Merchant A, Nitsch M. Factors influencing workplace health promotion intervention: a qualitative systematic review. Health Promot Int. 2017;32(5):831–9. doi: 10.1093/heapro/daw015 27006365

[26] Wierenga D, Engbers LH, Van Empelen P, Duijts S, Hildebrandt VH, Van Mechelen W. What is actually measured in process evaluations for worksite health promotion programs: a systematic review. BMC Public Health. 2013;13:1190. doi: 10.1186/1471-2458-13-1190 24341605 PMC3890539

[27] van de Ven D, Schuring M, Kouwenhoven-Pasmooij TA, Blom P, Burdorf A, Robroek SJW. Reach and effectiveness of a worksite health promotion program combining a preventive medical examination with motivational interviewing; a quasi-experimental study among workers in low socioeconomic position. BMC Public Health. 2023;23(1):2130. doi: 10.1186/s12889-023-16908-w 37904106 PMC10617210

[28] Robroek SJ, van Lenthe FJ, van Empelen P, Burdorf A. Determinants of participation in worksite health promotion programmes: a systematic review. Int J Behav Nutr Phys Act. 2009;6:26. doi: 10.1186/1479-5868-6-26 19457246 PMC2698926

[29] Bernstein A, Liang A, Luo F, Mann E, Shilts E, Serxner S. Financial incentives and employer-sponsored health activities. J Occup Environ Med. 2020;62(11). 10.1097/JOM.0000000000002003 32826553

[30] Volpp KG, Troxel AB, Pauly MV, Glick HA, Puig A, Asch DA, et al. A randomized, controlled trial of financial incentives for smoking cessation. N Engl J Med. 2009;360(7):699–709. doi: 10.1056/NEJMsa0806819 19213683

[31] Fraser DL, Fiore MC, Kobinsky K, Adsit R, Smith SS, Johnson ML, et al. A randomized trial of incentives for smoking treatment in Medicaid members. Am J Prev Med. 2017;53(6):754–63. doi: 10.1016/j.amepre.2017.08.027 29079405 PMC5978743

[32] Parks MJ, Hughes KD, Keller PA, Lachter RB, Kingsbury JH, Nelson CL, et al. Financial incentives and proactive calling for reducing barriers to tobacco treatment among socioeconomically disadvantaged women: a factorial randomized trial. Prev Med. 2019;129:105867. doi: 10.1016/j.ypmed.2019.105867 31634512

[33] Baker TB, Fraser DL, Kobinsky K, Adsit R, Smith SS, Khalil L, et al. A randomized controlled trial of financial incentives to low income pregnant women to engage in smoking cessation treatment: effects on post-birth abstinence. J Consult Clin Psychol. 2018;86(5):464–73. doi: 10.1037/ccp0000278 29389142

[34] Robertson L, Gendall P, Hoek J, Marsh L, McGee R. Perceptions of financial incentives for smoking cessation: a survey of smokers in a country with an endgame goal. Nicotine Tob Res. 2018;20(12):1481–8. doi: 10.1093/ntr/ntx268 29253215

[35] Berlin N, Goldzahl L, Bauld L, Hoddinott P, Berlin I. Public acceptability of financial incentives to reward pregnant smokers who quit smoking: a United Kingdom-France comparison. Eur J Health Econ. 2017. doi: 10.1007/s10198-017-0914-6 28646249 PMC5948294

[36] Hoskins K, Ulrich CM, Shinnick J, Buttenheim AM. Acceptability of financial incentives for health-related behavior change: an updated systematic review. Prev Med. 2019;126:105762. doi: 10.1016/j.ypmed.2019.105762 31271816

[37] Fernandez ME, ten Hoor GA, van Lieshout S, Rodriguez SA, Beidas RS, Parcel G. Implementation mapping: using intervention mapping to develop implementation strategies. Front Public Health. 2019;7. doi: 10.3389/fpubh.2019.00158 PMC6592155 31275915

[38] Glasgow RE, Harden SM, Gaglio B, Rabin B, Smith ML, Porter GC, et al. RE-AIM planning and evaluation framework: adapting to new science and practice with a 20-year review. Front Public Health. 2019;7:64. doi: 10.3389/fpubh.2019.00064 30984733 PMC6450067

[39] van den Brand FA, Nagelhout GE, Winkens B, Chavannes NH, van Schayck OCP, Evers SMAA. Cost-effectiveness and cost-utility analysis of a work-place smoking cessation intervention with and without financial incentives. Addiction. 2020;115(3):534–45. doi: 10.1111/add.14861 31849138 PMC7027826

[40] Donaldson A, Lloyd DG, Gabbe BJ, Cook J, Finch CF. We have the programme, what next? Planning the implementation of an injury prevention programme. Inj Prev. 2017;23(4):273–80. doi: 10.1136/injuryprev-2015-041737 26787739 PMC5537515

[41] Poole NL, Nagelhout GE, Magnée T, de Haan-Bouma LCI, Barendregt C, van Schayck OCP, et al. A qualitative study assessing how reach and participation can be improved in workplace smoking cessation programs. Tob Prev Cessat. 2023;9:07. doi: 10.18332/tpc/161589 36968254 PMC10037216

[42] van den Brand FA, Magnée T, de Haan-Bouma L, Barendregt C, Chavannes NH, van Schayck OCP, et al. Implementation of financial incentives for successful smoking cessation in real-life company settings: a qualitative needs assessment among employers. Int J Environ Res Public Health. 2019;16(24):5135. doi: 10.3390/ijerph16245135 31888195 PMC6950050

[43] Ajzen I. The theory of planned behavior. Organ Behav Hum Decis Process. 1991;50(2):179–211. doi: 10.1016/0749-5978(91)90020-T

[44] Bandura A. Health promotion by social cognitive means. Health Educ Behav. 2004;31(2):143–64. doi: 10.1177/1090198104263660 15090118

[45] Rosenstock IM. Historical origins of the health belief model. Health Educ Monogr. 1974;2(4):328–35. doi: 10.1177/109019817400200403 299611

[46] Kok G, Gottlieb NH, Peters G-JY, Mullen PD, Parcel GS, Ruiter RAC, et al. A taxonomy of behaviour change methods: an Intervention Mapping approach. Health Psychol Rev. 2016;10(3):297–312. doi: 10.1080/17437199.2015.1077155 26262912 PMC4975080

[47] Glasgow RE, Vogt TM, Boles SM. Evaluating the public health impact of health promotion interventions: the RE-AIM framework. Am J Public Health. 1999;89(9):1322–7. doi: 10.2105/ajph.89.9.1322 10474547 PMC1508772

[48] Steckler AB, Linnan L, Israel B. Process evaluation for public health interventions and research. San Francisco (CA): Jossey-Bass; 2002.

[49] Passey DG, Hammerback K, Huff A, Harris JR, Hannon PA. The role of managers in employee wellness programs: a mixed-methods study. Am J Health Promot. 2018;32(8):1697–705. doi: 10.1177/0890117118767785 29649896

[50] Greenberg KL, Donchin M, Leiter E, Zwas DR. Health ambassadors in the workplace: a health promotion intervention mobilizing middle managers and RE-AIM evaluation of outcomes. BMC Public Health. 2021;21(1):1585. doi: 10.1186/s12889-021-11609-8 34425815 PMC8383401

[51] Cosgrave E, Sheridan A, Murphy E, Blake M, Siersbaek R, Parker S, et al. Public attitudes to implementing financial incentives in stopsmoking services in Ireland. Tob Prev Cessat. 2023;9:09. doi: 10.18332/tpc/162364 37020632 PMC10068872

[52] van der Spek L, Breunis LJ, Scheffers-van Schayck T, Bauld L, Ista E, Been JV. Financial incentives for smoking cessation among (expectant) parents: a systematic review of facilitators and barriers to implementation. Tob Control. 2026;35(4):430–9. doi: 10.1136/tc-2024-059198 40262855 PMC13479641

[53] Siersbaek R, Kavanagh P, Ford J, Burke S, Parker S. How and why do financial incentives contribute to helping people stop smoking? A realist review. BMC Public Health. 2024;24(1):500. doi: 10.1186/s12889-024-17967-3 38365629 PMC10873947

[54] Breen RJ, Palmer MA, Frandsen M, Ferguson SG. Design of financial incentive programs for smoking cessation: a discrete choice experiment. Nicotine Tob Res. 2022;24(10):1661–8. doi: 10.1093/ntr/ntac042 35165733 PMC9575978

[55] van Wijk EC, Landais LL, Harting J. Understanding the multitude of barriers that prevent smokers in lower socioeconomic groups from accessing smoking cessation support: a literature review. Prev Med. 2019;123:143–51. doi: 10.1016/j.ypmed.2019.03.029 30902700

