# Supplementary material for: Development of an implementation strategy for a workplace smoking cessation program with financial incentives using Implementation Mapping
Source: PLoS One. 2026 Sep 18;21(9):e0336553. doi: 10.1371/journal.pone.0336553 (PMC13588387; doi:10.1371/journal.pone.0336553)
Supplement: Supplementary file 1 [file pone.0336553.s001.docx]

Supporting information 1. Matrices of change objectives for Reach, Adoption, Implementation and Maintenance to promote the implementation of smoking cessation group trainings with financial incentives in organizations.

| **Stage** | **Behavioral**  **Outcomes** | **Performance objectives** | **Change objectives** | | | | | |
| --- | --- | --- | --- | --- | --- | --- | --- | --- |
|  |  |  | **Determinants** | | | | | |
|  |  |  | **Attitude** | **Outcome expectations** | **Skills & self-efficacy** | **Knowledge** | **Normative beliefs** | **Perceived Barriers** |
| **Reach** | BO1 The employer actively raises awareness among employees with a lower SEP about the smoking cessation training and motivates them to participate | PO1.1 The employer will proactively communicate in-person with lower SEP employees regarding the smoking cessation training.  PO1.2 The employer will utilize diverse communication channels to inform all employees about the smoking cessation training. | A1.1 Demonstrates a positive attitude toward initiating in-person conversations with employees about smoking.  A1.2 Expresses the belief that an active, personal approach is essential for effective communication.  A1.3 Expresses the belief that discussing smoking with employees is a manageable and straightforward task. | OE1.1 Expresses the belief that the short advice is effective in increasing employee enrolment rates.  OE1.2 Expresses the belief that having a conversation with employees about smoking has a positive effect on the relationship with the employee.  OE1.3 Expresses the belief that having a conversation with employees about smoking will increase employee wellbeing.  OE1.4 Expresses the belief that having a conversation with employees about smoking will promote smoking cessation. | SS1.1 Expresses confidence in ability to approach employees in person to discuss smoking behavior.  SS1.2 Demonstrates the ability to persuade employees to participate in smoking cessation training.  SS1.3 Demonstrates the ability to explain the organization's commitment to promoting smoking cessation.  SS1.4 Demonstrates the ability to actively listen and respond without judgment during. conversations about smoking.  SS1.5 Expresses confidence in handling defensive reactions from employees. | K1.1 Lists the health benefits of quitting smoking.  K1.2 Identifies employee groups more likely to smoke.  K1.3 Explains the effectiveness of approaching employees in-person about smoking.  K1.4 Provides information on where employees can sign up for smoking cessation training or access support.  K1.5 Recognizes examples of effective and ineffective communication.  K1.6 Identifies appropriate times and places to discuss smoking cessation with employees.  K1.7 Articulates the organization's rationale for offering smoking cessation support.  K1.8 Lists appropriate responses to employees who react defensively. | NB1.1 Acknowledges that employees appreciate in-person discussions about smoking.  NB1.2 Recognizes that many employees who smoke are contemplating quitting.  NB1.3 Understands that employees value being informed about cessation training.  NB1.4 Acknowledges that employees, management, and other organizations see discussions about smoking cessation as part of good employer practice.  NB1.5 Recognizes that promoting employee health is considered an employer's responsibility by employees, management, and broader society.  NB1.6 Aware that society generally supports smoking cessation initiatives. | PB1.1 Aware of personal barriers to initiating in-person discussions about smoking.  PB1.2 Recognizes personal discomfort when addressing smoking behaviors with employees.  PB1.3 Understands that some employees may be reluctant to discuss their smoking habits.  PB1.4 Anticipates potential defensive reactions from employees. |

| **Stage** | **Behavioral**  **Outcomes** | **Performance objectives** | **Change objectives** | | | | | |
| --- | --- | --- | --- | --- | --- | --- | --- | --- |
|  |  |  | **Determinants** | | | | | |
|  |  |  | **Attitude** | **Outcome expectations** | **Skills & self-efficacy** | **Knowledge** | **Normative beliefs** | **Perceived Barriers** |
| **Adoption** | BO2 The employer adopts the intervention by formalizing an agreement with a training provider for smoking cessation. | PO2.1: The employer commits to offering a smoking cessation group training.  PO2.2 The employer decides to incorporate financial incentives to encourage smoking cessation success. | A2.1 Expresses a positive attitude toward implementing a smoking cessation training.  A2.2 Expresses a positive attitude toward financial incentives for smoking cessation success, viewing them as fair and beneficial. | OE2.1 Expresses the belief that organizing a smoking cessation training will help employees quit smoking.  OE2.2 Expresses the belief that adding financial incentives to the training increases smoking cessation rates. | SS2.1 Demonstrates the ability to persuade the organization to arrange the smoking cessation training.  SS2.2 Expresses confidence in convincing the organization to use financial incentives. | K2.1 Demonstrates knowledge of the overall benefits of combining smoking cessation training with financial incentives for all employees.  K2.2 Understands the key characteristics of effective financial incentives. | NB2.1 Recognizes that offering smoking cessation support is seen as a mark of a responsible employer.  NB2.2 Acknowledges that both smoking and non-smoking employees support the use of financial incentives for smoking cessation. | PB2.1 Anticipates that some within the organization may not support offering a smoking cessation training.  PB2.2 Acknowledges that some stakeholders may object to the use of financial incentives. |

| **Stage** | **Behavioral**  **Outcomes** | **Performance objectives** | **Change objectives** | | | | | |
| --- | --- | --- | --- | --- | --- | --- | --- | --- |
|  |  |  | **Determinants** | | | | | |
|  |  |  | **Attitude** | **Outcome expectations** | **Skills & self-efficacy** | **Knowledge** | **Normative beliefs** | **Perceived Barriers** |
| **Implementation** | BO3 The employer implements the intervention within the organization (or specific department). | PO3.1: The employer organizes the smoking cessation training, ensuring accessibility for lower SEP employees by offering it in-house during working hours or reimbursing time spent.  PO3.2: The employer selects an effective type and schedule for the financial incentives.  PO3.3: The employer actively recruits employees to participate in the intervention. | A3.1 Expresses a positive attitude toward organizing an accessible smoking cessation training  A3.2 Expresses a positive attitude toward offering financial incentives for smoking cessation success | OE3.1 Expresses the belief that employees will participate in the smoking cessation training  OE3.2 Expresses the belief that combining smoking cessation training with financial incentives will lead to higher quit rates.  OE3.3 Expresses the belief that implementing the intervention will be cost-effective for the organization. | SS3.1 Demonstrates the skills to plan, promote and execute the smoking cessation training and financial incentives | K3.1 Understands the necessary steps to arrange and implement the smoking cessation training  K3.2 Demonstrates knowledge of how to effectively set up financial incentives | NB3.1 Recognizes that there is an expectation to actively organize and promote the intervention. | PB3.1 Recognizes that it is needed to prioritize and allocate sufficient time to ensure successful implementation of the intervention. |

| **Stage** | **Behavioral**  **Outcomes** | **Performance objectives** | **Change objectives** | | | | | |
| --- | --- | --- | --- | --- | --- | --- | --- | --- |
|  |  |  | **Determinants** | | | | | |
|  |  |  | **Attitude** | **Outcome expectations** | **Skills & self-efficacy** | **Knowledge** | **Normative beliefs** | **Perceived Barriers** |
| **Maintenance** | BO4  The employer ensures that the smoking cessation training with financial incentives is provided at least a second time as indicated by him or her signing a second contract with a provider of the training. | PO4.1: The employer agrees to provide the smoking cessation training with financial incentives for a second round within the organization. | A4.1 Expresses the belief that ongoing smoking cessation support is essential for both new employees and those who may need support in the future. | OE4.1 Expresses the expectation that maintaining the smoking cessation support with financial incentives will result in more employees successfully quitting smoking. | - | - | NB4.1 Recognizes that organizations are expected to continue offering smoking cessation support to their employees. | PB4.1 Acknowledges that key barriers to sustaining the intervention include a lack of priority, time and budget. |
