# Supplementary material for: Development of an implementation strategy for a workplace smoking cessation program with financial incentives using Implementation Mapping
Source: PLoS One. 2026 Sep 18;21(9):e0336553. doi: 10.1371/journal.pone.0336553 (PMC13588387; doi:10.1371/journal.pone.0336553)
Supplement: Supplementary file 3 [file pone.0336553.s004.docx]

**Response to reviewers**

Manuscript: PONE-D-25-57654

Development of an implementation strategy for a workplace smoking cessation program with financial incentives using Implementation Mapping

- Merge the conclusion and implication into one section and do not repeat any results.

Authors: We have merged the conclusion and implication section and removed overlap.

- Bring the context high up in the introduction, more or less, like a compelling case without the title "Tobacco smoking in the Netherlands."

Authors: We have integrated this paragraph into the main Introduction instead of in a separate Box.

- Split Table 3 into three tables - reach; adoption; and implementation and maintenance.

Authors: We have split Table 3 as suggested.

- Reduce the description of the materials developed; keep them simple and succinct.

Authors: We have reduced the description of the materials.
