# Supplementary material for: Development of an implementation strategy for a workplace smoking cessation program with financial incentives using Implementation Mapping
Source: PLoS One. 2026 Sep 18;21(9):e0336553. doi: 10.1371/journal.pone.0336553 (PMC13588387; doi:10.1371/journal.pone.0336553)
Supplement: Peer review history

# Peer review: Decision Letter 0

**DOI:** 10.1371/journal.pone.0336553.r001

**Authors:** Daniel Ogbuabor

3 Mar 2026

Dear Dr. van den Brand,

Thank you for submitting your manuscript to PLOS ONE. After careful consideration, we feel that it has merit but does not fully meet PLOS ONE’s publication criteria as it currently stands. Therefore, we invite you to submit a revised version of the manuscript that addresses the points raised during the review process.

==============================

**ACADEMIC EDITOR:**

- address the comments of the reviewers, point-by-point.

Please submit your revised manuscript by Apr 17 2026 11:59PM. If you will need more time than this to complete your revisions, please reply to this message or contact the journal office at plosone@plos.org. When you're ready to submit your revision, log on to https://www.editorialmanager.com/pone/ and select the 'Submissions Needing Revision' folder to locate your manuscript file.

- A letter that responds to each point raised by the academic editor and reviewer(s). You should upload this letter as a separate file labeled 'Response to Reviewers'.
- A marked-up copy of your manuscript that highlights changes made to the original version. You should upload this as a separate file labeled 'Revised Manuscript with Track Changes'.
- An unmarked version of your revised paper without tracked changes. You should upload this as a separate file labeled 'Manuscript'.

If you would like to make changes to your financial disclosure, please include your updated statement in your cover letter. Guidelines for resubmitting your figure files are available below the reviewer comments at the end of this letter.

If applicable, we recommend that you deposit your laboratory protocols in protocols.io to enhance the reproducibility of your results. Protocols.io assigns your protocol its own identifier (DOI) so that it can be cited independently in the future. For instructions see: https://journals.plos.org/plosone/s/submission-guidelines#loc-laboratory-protocols. Additionally, PLOS ONE offers an option for publishing peer-reviewed Lab Protocol articles, which describe protocols hosted on protocols.io. Read more information on sharing protocols at https://plos.org/protocols?utm_medium=editorial-email&utm_source=authorletters&utm_campaign=protocols.

We look forward to receiving your revised manuscript.

Kind regards,

Daniel Chukwuemeka Ogbuabor, Ph.D., M.D.

Academic Editor

PLOS One

Journal Requirements:

When submitting your revision, we need you to address these additional requirements.

1. Please ensure that your manuscript meets PLOS ONE's style requirements, including those for file naming. The PLOS ONE style templates can be found at

https://journals.plos.org/plosone/s/file?id=wjVg/PLOSOne_formatting_sample_main_body.pdf and

https://journals.plos.org/plosone/s/file?id=ba62/PLOSOne_formatting_sample_title_authors_affiliations.pdf

2. Your ethics statement should only appear in the Methods section of your manuscript. If your ethics statement is written in any section besides the Methods, please delete it from any other section.

3. We note that you have indicated that there are restrictions to data sharing for this study. For studies involving human research participant data or other sensitive data, we encourage authors to share de-identified or anonymized data. However, when data cannot be publicly shared for ethical reasons, we allow authors to make their data sets available upon request. For information on unacceptable data access restrictions, please see http://journals.plos.org/plosone/s/data-availability#loc-unacceptable-data-access-restrictions.

Before we proceed with your manuscript, please address the following prompts:

a) If there are ethical or legal restrictions on sharing a de-identified data set, please explain them in detail (e.g., data contain potentially identifying or sensitive patient information, data are owned by a third-party organization, etc.) and who has imposed them (e.g., a Research Ethics Committee or Institutional Review Board, etc.). Please also provide contact information for a data access committee, ethics committee, or other institutional body to which data requests may be sent.

b) If there are no restrictions, please upload the minimal anonymized data set necessary to replicate your study findings to a stable, public repository and provide us with the relevant URLs, DOIs, or accession numbers. Please see http://www.bmj.com/content/340/bmj.c181.long for guidelines on how to de-identify and prepare clinical data for publication. For a list of recommended repositories, please see https://journals.plos.org/plosone/s/recommended-repositories. You also have the option of uploading the data as Supporting Information files, but we would recommend depositing data directly to a data repository if possible.

Please update your Data Availability statement in the submission form accordingly.

4. Please amend your list of authors on the manuscript to ensure that each author is linked to an affiliation. Authors’ affiliations should reflect the institution where the work was done (if authors moved subsequently, you can also list the new affiliation stating “current affiliation:….” as necessary).

5. Please include a separate caption for each figure in your manuscript.

6. Please ensure that you refer to Figure 1 in your text as, if accepted, production will need this reference to link the reader to the figure.

7. We note you have included a table to which you do not refer in the text of your manuscript. Please ensure that you refer to Table 4 in your text; if accepted, production will need this reference to link the reader to the Table.

8. If the reviewer comments include a recommendation to cite specific previously published works, please review and evaluate these publications to determine whether they are relevant and should be cited. There is no requirement to cite these works unless the editor has indicated otherwise.

Additional Editor Comments:

Be sure to address the comments of the reviewers.

[Note: HTML markup is below. Please do not edit.]

Reviewers' comments:

Reviewer's Responses to Questions

**Comments to the Author**

1. Is the manuscript technically sound, and do the data support the conclusions?

Reviewer #1: Yes

Reviewer #2: Yes

**********

2. Has the statistical analysis been performed appropriately and rigorously?

Reviewer #1: Yes

Reviewer #2: N/A

**********

3. Have the authors made all data underlying the findings in their manuscript fully available?

The PLOS Data policy

Reviewer #1: Yes

Reviewer #2: No

**********

4. Is the manuscript presented in an intelligible fashion and written in standard English?

Reviewer #1: Yes

Reviewer #2: Yes

**********

Reviewer #1: This is an interesting and important study with clear design and procedures described. I would suggest authors to give a little bit more information about the context (companies) so to be useful to finding generalization.

Reviewer #2: PONE-D-25-57654

Development of an implementation strategy for a workplace smoking cessation program

with financial incentives using Implementation Mapping

Reviewer comments

Introduction:

- Please cite the recent systematic review which shows that financial incentives to stop smoking can be upto 50% effective in achieving smoking cessation: https://pubmed.ncbi.nlm.nih.gov/39799985/

- Authors state ‘offering financial incentives has been shown to increase participation rates’ – it would be good to see by how much does participation increase, and among which populations e.g. higher SEP or lower SEP, younger or older individuals etc.

Setting and target group

- More detail about the number of companies involved and the average number of employees in these companies is needed. Was this a nationwide approach or limited to certain regions / areas in the Netherlands? Also, how were the target groups selected, was there a sampling approach used to identify the companies? What about consent from these companies to participate – was this received and from who? E.g. company directors, employees directly?

Implementation planning group

- A flow diagram summarising the participation of various individuals would be useful to illustrate how the co-design and interview process was carried out.

- The employees involved, were they from different SEP / different companies?

- Clarity on whether meetings were mostly in-person, online, or a combination of both, and with which groups involved, would be useful for additional detail and context.

Results

Task 1: Conduct a needs assessment and identify adopters and implementers

- The first paragraph in this sub-section should be included in the methods section as it describes what was done. The results under Task 1 should simply report what is outlined in ‘Needs assessment amongst employers and employees’

- Similarly for the remaining Tasks outlined in the results, some components can be moved to the Methods section to explain to the reader how each task was performed, with key results from each task limited and summarised in the Results section.

- Table 1. Clarify that the key actions were a result of what came through post interviews etc.

- Table 2. Is there a better way to summarise the matrices e.g. use a flow diagram and moving the table to an appendix? In its current form, Table 1 is very busy and can be quite difficult to interpret

- Table 4 could be included as an appendix

Discussion

- The discussion is lacking comparability of this research in relation to similar research reported across other contexts / countries. Please highlight how these findings compare to other published evidence.

- What are the key implications of this research?

- What about other countries looking to adapt a similar IM approach? i.e. how can these results be generalisable to other contexts/ countries? Is this study very specific to the context across workplaces in the Netherlands? Are there any improvements in this research that could be made?

**********

what does this mean?). If published, this will include your full peer review and any attached files.

If you choose “no”, your identity will remain anonymous but your review may still be made public.

**Do you want your identity to be public for this peer review?**  For information about this choice, including consent withdrawal, please see our Privacy Policy

Reviewer #1: No

Reviewer #2: No

**********

[NOTE: If reviewer comments were submitted as an attachment file, they will be attached to this email and accessible via the submission site. Please log into your account, locate the manuscript record, and check for the action link "View Attachments". If this link does not appear, there are no attachment files.]

To ensure your figures meet our technical requirements, please review our figure guidelines: https://journals.plos.org/plosone/s/figures

You may also use PLOS’s free figure tool, NAAS, to help you prepare publication quality figures: https://journals.plos.org/plosone/s/figures#loc-tools-for-figure-preparation.

NAAS will assess whether your figures meet our technical requirements by comparing each figure against our figure specifications.

# Author comment: Author response to Decision Letter 1

**DOI:** 10.1371/journal.pone.0336553.r002

7 May 2026

Response to reviewers

Manuscript: PONE-D-25-57654

Development of an implementation strategy for a workplace smoking cessation program with financial incentives using Implementation Mapping

ACADEMIC EDITOR:

Thank you for considering our manuscript for publication in PLOS ONE. Please find our response to all comments below.

Journal Requirements:

When submitting your revision, we need you to address these additional requirements.

1. Please ensure that your manuscript meets PLOS ONE's style requirements, including those for file naming. The PLOS ONE style templates can be found at

https://journals.plos.org/plosone/s/file?id=wjVg/PLOSOne_formatting_sample_main_body.pdf and

https://journals.plos.org/plosone/s/file?id=ba62/PLOSOne_formatting_sample_title_authors_affiliations.pdf

Authors: we have made sure that the manuscript meets the style requirements.

2. Your ethics statement should only appear in the Methods section of your manuscript. If your ethics statement is written in any section besides the Methods, please delete it from any other section.

Authors: the ethics statement is now only written in the Methods section of our manuscript

3. We note that you have indicated that there are restrictions to data sharing for this study. For studies involving human research participant data or other sensitive data, we encourage authors to share de-identified or anonymized data. However, when data cannot be publicly shared for ethical reasons, we allow authors to make their data sets available upon request. For information on unacceptable data access restrictions, please see http://journals.plos.org/plosone/s/data-availability#loc-unacceptable-data-access-restrictions.

Authors: we have now added underlying data in Supporting Information file 1. We do not have any other underlying data set.

Before we proceed with your manuscript, please address the following prompts:

a) If there are ethical or legal restrictions on sharing a de-identified data set, please explain them in detail (e.g., data contain potentially identifying or sensitive patient information, data are owned by a third-party organization, etc.) and who has imposed them (e.g., a Research Ethics Committee or Institutional Review Board, etc.). Please also provide contact information for a data access committee, ethics committee, or other institutional body to which data requests may be sent.

b) If there are no restrictions, please upload the minimal anonymized data set necessary to replicate your study findings to a stable, public repository and provide us with the relevant URLs, DOIs, or accession numbers. Please see http://www.bmj.com/content/340/bmj.c181.long for guidelines on how to de-identify and prepare clinical data for publication. For a list of recommended repositories, please see https://journals.plos.org/plosone/s/recommended-repositories. You also have the option of uploading the data as Supporting Information files, but we would recommend depositing data directly to a data repository if possible.

Please update your Data Availability statement in the submission form accordingly.

Authors: we have uploaded our data as Supporting Information file 1.

4. Please amend your list of authors on the manuscript to ensure that each author is linked to an affiliation. Authors’ affiliations should reflect the institution where the work was done (if authors moved subsequently, you can also list the new affiliation stating “current affiliation:….” as necessary).

Authors: we have linked the authors to their affiliation.

5. Please include a separate caption for each figure in your manuscript.

Authors: we have added the caption for Figure 1:

Figure 1. Sample of developed materials for the implementation of the smoking cessation intervention. (A) Recruitment poster, (B) Communication factsheet page, (C) Logo, (D) Implementation planning guide cover, (E) Website page (F) Video demonstrating effective communication, (G) Webinar recording.

6. Please ensure that you refer to Figure 1 in your text as, if accepted, production will need this reference to link the reader to the figure.

Authors: we have added the reference to Figure 1.

7. We note you have included a table to which you do not refer in the text of your manuscript. Please ensure that you refer to Table 4 in your text; if accepted, production will need this reference to link the reader to the Table.

Authors: we have added the reference to Table 4 in our text.

8. If the reviewer comments include a recommendation to cite specific previously published works, please review and evaluate these publications to determine whether they are relevant and should be cited. There is no requirement to cite these works unless the editor has indicated otherwise.

Authors: thank you, we have done this.

Additional Editor Comments:

Be sure to address the comments of the reviewers.

Authors: thank you, we have responded to the comments of the reviewers below.

[Note: HTML markup is below. Please do not edit.]

Reviewers' comments:

Reviewer's Responses to Questions

Comments to the Author

1. Is the manuscript technically sound, and do the data support the conclusions?

The manuscript must describe a technically sound piece of scientific research with data that supports the conclusions. Experiments must have been conducted rigorously, with appropriate controls, replication, and sample sizes. The conclusions must be drawn appropriately based on the data presented.

Reviewer #1: Yes

Reviewer #2: Yes

________________________________________

2. Has the statistical analysis been performed appropriately and rigorously?

Reviewer #1: Yes

Reviewer #2: N/A

________________________________________

3. Have the authors made all data underlying the findings in their manuscript fully available?

The PLOS Data policy requires authors to make all data underlying the findings described in their manuscript fully available without restriction, with rare exception (please refer to the Data Availability Statement in the manuscript PDF file). The data should be provided as part of the manuscript or its supporting information, or deposited to a public repository. For example, in addition to summary statistics, the data points behind means, medians and variance measures should be available. If there are restrictions on publicly sharing data—e.g. participant privacy or use of data from a third party—those must be specified.

Reviewer #1: Yes

Reviewer #2: No

________________________________________

4. Is the manuscript presented in an intelligible fashion and written in standard English?

PLOS ONE does not copyedit accepted manuscripts, so the language in submitted articles must be clear, correct, and unambiguous. Any typographical or grammatical errors should be corrected at revision, so please note any specific errors here.

Reviewer #1: Yes

Reviewer #2: Yes

________________________________________

5. Review Comments to the Author

Please use the space provided to explain your answers to the questions above. You may also include additional comments for the author, including concerns about dual publication, research ethics, or publication ethics. (Please upload your review as an attachment if it exceeds 20,000 characters)

Reviewer #1: This is an interesting and important study with clear design and procedures described. I would suggest authors to give a little bit more information about the context (companies) so to be useful to finding generalization.

Authors: thank you for reviewing our manuscript and your helpful suggestion. We have added more information about the context of companies in the Netherlands to the Introduction (page 6): “In the Netherlands, a workplace smoking ban has been in force since 1 January 2004, giving employees the right to a smoke-free workplace. This legislation has subsequently been strengthened, including the prohibition of indoor smoking rooms from 1 January 2021. The ban also applies to the hospitality sector and covers all tobacco products as well as e-cigarettes. In addition, the Dutch public campaign Smoke-Free Generation (Dutch: Rookvrije Generatie) promotes smoking cessation through the workplace by encouraging companies to implement smoke-free workplaces and grounds, and support employees in quitting, for example through cessation support or referral to professional services.”

To compensate for the added text, we have also made some changes to the Introduction to reduce the word count.

Reviewer #2: PONE-D-25-57654

Development of an implementation strategy for a workplace smoking cessation program

with financial incentives using Implementation Mapping

Reviewer comments

Authors: thank you for reviewing our manuscript and your helpful suggestions. Please find our responses below.

Introduction:

- Please cite the recent systematic review which shows that financial incentives to stop smoking can be upto 50% effective in achieving smoking cessation: https://pubmed.ncbi.nlm.nih.gov/39799985/

Authors: thank you for noticing this: we already referred to this systematic review in our Introduction, but we also, by mistake, referred to the previous (2019) version of this review. We have now updated this and only refer to the updated 2025 version.

- Authors state ‘offering financial incentives has been shown to increase participation rates’ – it would be good to see by how much does participation increase, and among which populations e.g. higher SEP or lower SEP, younger or older individuals etc.

Authors: We have added a more specific description to the introduction (page 5/6):

“In a large study, incentives increased employee enrollment in a smoking cessation program from 5.4% to 15.4% and program completion from 2.5% to 10.8%. (Volpp et al., 2009) Similar positive effects on smoking treatment engagement have been reported in lower-SEP populations.”

Setting and target group

- More detail about the number of companies involved and the average number of employees in these companies is needed. Was this a nationwide approach or limited to certain regions / areas in the Netherlands? Also, how were the target groups selected, was there a sampling approach used to identify the companies? What about consent from these companies to participate – was this received and from who? E.g. company directors, employees directly?

Authors: the paper describes merely the development of the implementation strategy, and therefore there was no implementation yet. We have tried to describe this more clear on page 7, and we have added more details: “In this study, we followed the Implementation Mapping process from January 2019 to September 2021 to develop an implementation strategy for a smoking cessation group training program with financial incentives that could be used by companies throughout the Netherlands. The strategy was specifically intended for implementation in companies with a high proportion of lower-SEP employees. We invited employers and employees from our professional networks to participate in the implementation planning group, including large organizations in the sheltered employment, healthcare, and public safety sectors, located across the Netherlands.”

Implementation planning group

- A flow diagram summarising the participation of various individuals would be useful to illustrate how the co-design and interview process was carried out.

- The employees involved, were they from different SEP / different companies?

- Clarity on whether meetings were mostly in-person, online, or a combination of both, and with which groups involved, would be useful for additional detail and context.

Authors: due to the already high number of Tables/figures in our manuscript, we believe there is little space for including and extra figure. We have added the suggested information (page 8): “two employees from different companies who smoke(d) (one moderate and one lower-SEP)” and “The first meeting was held in person; due to the COVID-19 pandemic, all subsequent meetings took place online via videoconference.”

Results

Task 1: Conduct a needs assessment and identify adopters and implementers

- The first paragraph in this sub-section should be included in the methods section as it describes what was done. The results under Task 1 should simply report what is outlined in ‘Needs assessment amongst employers and employees’

- Similarly for the remaining Tasks outlined in the results, some components can be moved to the Methods section to explain to the reader how each task was performed, with key results from each task limited and summarised in the Results section.

Thank you for this helpful suggestion. We agree that several parts of the Task descriptions were more methodological than result-oriented. We have therefore revised the manuscript to improve the distinction between Methods and Results. Specifically, we moved the first paragraph under Task 1 to the Methods section, under the subheading Implementation Mapping. In addition, we reviewed the descriptions of the remaining Tasks and relocated methodological details where needed to the Methods section, while keeping the Results section focused on the main findings and a concise summary of the outcomes for each Task. Because we made extensive changes to these paragraphs, we would like to refer to the manuscript with tracked changes rather than copy the new text here.

- Table 1. Clarify that the key actions were a result of what came through post interviews etc.

Authors: we have improved the description of Table 1: “Table 1. Key actions required to improve the implementation of workplace smoking cessation group trainings with financial incentives, as identified from qualitative interviews with employers and employees conducted as part of the needs assessment.”

- Table 2. Is there a better way to summarise the matrices e.g. use a flow diagram and moving the table to an appendix? In its current form, Table 1 is very busy and can be quite difficult to interpret

Authors: Thank you for this helpful comment. We agree that the table was busy in its original form and could be difficult to interpret. To improve clarity, we have replaced the detailed Table in the main text with a simpler visual summary (Fig2) using a flow diagram, and we have moved the full table to the Supporting Material (S1).

- Table 4 could be included as an appendix

Authors: we are certainly willing to include Table 4 in the appendix instead. However, we would prefer to keep it in the manuscript, as it provides an overview of the tangible materials developed as part of the implementation strategy. This is important because the study aimed not only to identify implementation needs and strategies, but also to translate these into concrete products to support adoption and implementation in practice. Including this table therefore strengthens the transparency, reproducibility, and practical relevance of the paper.

Discussion

- The discussion is lacking comparability of this research in relation to similar research reported across other contexts / countries. Please highlight how these findings compare to other published evidence.

- What are the key implications of this research?

- What about other countries looking to adapt a similar IM approach? i.e. how can these results be generalisable to other contexts/ countries? Is this study very specific to the context across workplaces in the Netherlands? Are there any improvements in this research that could be made?

Thank you for this helpful comment. We agree that the Discussion needed a clearer positioning of our findings in relation to international evidence, as well as a more explicit reflection on implications and transferability. We have therefore revised the Discussion accordingly. We now compare our findings with published evidence from other workplace smoking cessation studies and reviews. We also state the implications of this research more explicitly. We highlight that successful implementation of workplace smoking cessation interventions requires not only evidence of effectiveness, but also a systematic, stakeholder-informed implementation strategy that addresses reach, adoption, and practical implementation barriers. In addition, we added text on transferability to other countries. We now dis

Attachment Submitted filename: Response to reviewers.docx

# Editor report: Decision Letter 1

**DOI:** 10.1371/journal.pone.0336553.r003

**Authors:** Daniel Ogbuabor

1 Jun 2026

Dear Dr. van den Brand,

Thank you for submitting your manuscript to PLOS ONE. After careful consideration, we feel that it has merit but does not fully meet PLOS ONE’s publication criteria as it currently stands. Therefore, we invite you to submit a revised version of the manuscript that addresses the points raised during the review process.

- Merge the conclusion and implication into one section and do not repeat any results.
- Bring the context high up in the introduction, more or less, like a compelling case without the title "Tobacco smoking in the Netherlands."
- Split Table 3 into three tables - reach; adoption; and implementation and maintenance.
- Reduce the description of the materials developed; keep them simple and succinct.

plosone@plos.org. When you're ready to submit your revision, log on to https://www.editorialmanager.com/pone/ and select the 'Submissions Needing Revision' folder to locate your manuscript file.

- A letter that responds to each point raised by the academic editor and reviewer(s). You should upload this letter as a separate file labeled 'Response to Reviewers'.
- A marked-up copy of your manuscript that highlights changes made to the original version. You should upload this as a separate file labeled 'Revised Manuscript with Track Changes'.
- An unmarked version of your revised paper without tracked changes. You should upload this as a separate file labeled 'Manuscript'.

If you would like to make changes to your financial disclosure, please include your updated statement in your cover letter. Guidelines for resubmitting your figure files are available below the reviewer comments at the end of this letter.

If applicable, we recommend that you deposit your laboratory protocols in protocols.io to enhance the reproducibility of your results. Protocols.io assigns your protocol its own identifier (DOI) so that it can be cited independently in the future. For instructions see: https://journals.plos.org/plosone/s/submission-guidelines#loc-laboratory-protocols. Additionally, PLOS ONE offers an option for publishing peer-reviewed Lab Protocol articles, which describe protocols hosted on protocols.io. Read more information on sharing protocols at https://plos.org/protocols?utm_medium=editorial-email&utm_source=authorletters&utm_campaign=protocols.

As the corresponding author, your ORCID iD is verified in the submission system and will appear in the published article. PLOS supports the use of ORCID, and we encourage all coauthors to register for an ORCID iD and use it as well. Please encourage your coauthors to verify their ORCID iD within the submission system before final acceptance, as unverified ORCID iDs will not appear in the published article. *Only*  the individual author can complete the verification step; PLOS staff *cannot*  verify ORCID iDs on behalf of authors.

We look forward to receiving your revised manuscript.

Kind regards,

Daniel Chukwuemeka Ogbuabor, Ph.D., M.D.

Academic Editor

PLOS One

Journal Requirements:

If the reviewer comments include a recommendation to cite specific previously published works, please review and evaluate these publications to determine whether they are relevant and should be cited. There is no requirement to cite these works unless the editor has indicated otherwise.

Please review your reference list to ensure that it is complete and correct. If you have cited papers that have been retracted, please include the rationale for doing so in the manuscript text, or remove these references and replace them with relevant current references. Any changes to the reference list should be mentioned in the rebuttal letter that accompanies your revised manuscript. If you need to cite a retracted article, indicate the article’s retracted status in the References list and also include a citation and full reference for the retraction notice.

Additional Editor Comments:

1. Merge the conclusion and implication into one section and do not repeat any results.

2. The context "Tobacco smoking in the Netherlands" is hanging in the introduction, positioned after the aim of the study. This context should come high up in the introduction, more or less, like a compelling case.

3. Split Table 3 into three tables - reach; adoption; and implementation and maintenance.

4. Reduce the description of the materials developed; keep them simple and succinct.

[Note: HTML markup is below. Please do not edit.]

[NOTE: If reviewer comments were submitted as an attachment file, they will be attached to this email and accessible via the submission site. Please log into your account, locate the manuscript record, and check for the action link "View Attachments". If this link does not appear, there are no attachment files.]

To ensure your figures meet our technical requirements, please review our figure guidelines: https://journals.plos.org/plosone/s/figures

You may also use PLOS’s free figure tool, NAAS, to help you prepare publication quality figures: https://journals.plos.org/plosone/s/figures#loc-tools-for-figure-preparation.

NAAS will assess whether your figures meet our technical requirements by comparing each figure against our figure specifications.

# Author comment: Author response to Decision Letter 2

**DOI:** 10.1371/journal.pone.0336553.r004

9 Jun 2026

Response to reviewers

Manuscript: PONE-D-25-57654

Development of an implementation strategy for a workplace smoking cessation program with financial incentives using Implementation Mapping

• Merge the conclusion and implication into one section and do not repeat any results.

Authors: We have merged the conclusion and implication section and removed overlap.

• Bring the context high up in the introduction, more or less, like a compelling case without the title "Tobacco smoking in the Netherlands."

Authors: We have integrated this paragraph into the main Introduction instead of in a separate Box.

• Split Table 3 into three tables - reach; adoption; and implementation and maintenance.

Authors: We have split Table 3 as suggested.

• Reduce the description of the materials developed; keep them simple and succinct.

Authors: We have reduced the description of the materials.

Attachment Submitted filename: Response to reviewers 9-6-2026.docx

# Editor report: Decision Letter 2

**DOI:** 10.1371/journal.pone.0336553.r005

**Authors:** Daniel Ogbuabor

10 Jun 2026

Development of an implementation strategy for a workplace smoking cessation program with financial incentives using Implementation Mapping

PONE-D-25-57654R2

Dear Dr. Floor van den Brand,

We’re pleased to inform you that your manuscript has been judged scientifically suitable for publication and will be formally accepted for publication once it meets all outstanding technical requirements.

Within one week, you’ll receive an e-mail detailing the required amendments. When these have been addressed, you’ll receive a formal acceptance letter and your manuscript will be scheduled for publication.

An invoice will be generated when your article is formally accepted. Please note, if your institution has a publishing partnership with PLOS and your article meets the relevant criteria, all or part of your publication costs will be covered. Please make sure your user information is up-to-date by logging into Editorial Manager at Editorial Manager® and clicking the ‘Update My Information' link at the top of the page. For questions related to billing, please contact billing support.

If your institution or institutions have a press office, please notify them about your upcoming paper to help maximize its impact. If they’ll be preparing press materials, please inform our press team as soon as possible -- no later than 48 hours after receiving the formal acceptance. Your manuscript will remain under strict press embargo until 2 pm Eastern Time on the date of publication. For more information, please contact onepress@plos.org.

Kind regards,

Daniel Chukwuemeka Ogbuabor, Ph.D., M.D.

Academic Editor

PLOS One

Additional Editor Comments (optional):

Reviewers' comments:

# Editor report: Acceptance letter

**DOI:** 10.1371/journal.pone.0336553.r006

**Authors:** Daniel Ogbuabor

PONE-D-25-57654R2

PLOS One

Dear Dr. van den Brand,

I'm pleased to inform you that your manuscript has been deemed suitable for publication in PLOS One. Congratulations! Your manuscript is now being handed over to our production team.

At this stage, our production department will prepare your paper for publication. This includes ensuring the following:

* All references, tables, and figures are properly cited

* All relevant supporting information is included in the manuscript submission,

* There are no issues that prevent the paper from being properly typeset

You will receive further instructions from the production team, including instructions on how to review your proof when it is ready. Please keep in mind that we are working through a large volume of accepted articles, so please give us a few days to review your paper and let you know the next and final steps.

Lastly, if your institution or institutions have a press office, please let them know about your upcoming paper now to help maximize its impact. If they'll be preparing press materials, please inform our press team within the next 48 hours. Your manuscript will remain under strict press embargo until 2 pm Eastern Time on the date of publication. For more information, please contact onepress@plos.org.

You will receive an invoice from PLOS for your publication fee after your manuscript has reached the completed accept phase. If you receive an email requesting payment before acceptance or for any other service, this may be a phishing scheme. Learn how to identify phishing emails and protect your accounts at https://explore.plos.org/phishing.

If we can help with anything else, please email us at customercare@plos.org.

Thank you for submitting your work to PLOS One and supporting open access.

Kind regards,

PLOS One Editorial Office Staff

on behalf of

Prof. Daniel Chukwuemeka Ogbuabor

Academic Editor

PLOS One
